# Ecological connectivity in the three-dimensional urban green volume using waveform airborne lidar

**DOI:** 10.1038/srep45571

**Published:** 2017-04-06

**Authors:** Stefano Casalegno, Karen Anderson, Daniel T. C. Cox, Steven Hancock, Kevin J. Gaston

**Affiliations:** 1 Environment & Sustainability Institute, University of Exeter, Penryn, Cornwall TR10 9EZ, UK; 2 Department of Geographical Sciences, University of Maryland, 2181 LeFrak Hall, College Park, MD 20740, USA

## Abstract

The movements of organisms and the resultant flows of ecosystem services are strongly shaped by landscape connectivity. Studies of urban ecosystems have relied on two-dimensional (2D) measures of greenspace structure to calculate connectivity. It is now possible to explore three-dimensional (3D) connectivity in urban vegetation using waveform lidar technology that measures the full 3D structure of the canopy. Making use of this technology, here we evaluate urban greenspace 3D connectivity, taking into account the full vertical stratification of the vegetation. Using three towns in southern England, UK, all with varying greenspace structures, we describe and compare the structural and functional connectivity using both traditional 2D greenspace models and waveform lidar-generated vegetation strata (namely, grass, shrubs and trees). Measures of connectivity derived from 3D greenspace are lower than those derived from 2D models, as the latter assumes that all vertical vegetation strata are connected, which is rarely true. Fragmented landscapes that have more complex 3D vegetation showed greater functional connectivity and we found highest 2D to 3D functional connectivity biases for short dispersal capacities of organisms (6 m to 16 m). These findings are particularly pertinent in urban systems where the distribution of greenspace is critical for delivery of ecosystem services.

Connectivity, the degree to which the movement of organisms is facilitated or impeded across a landscape, is key to how that landscape functions ecologically^1^. It is a particularly critical issue in urban areas because these typically comprise very large numbers of, often small, habitat patches separated to varying degrees by impermeable surfaces and barriers (i.e. buildings and roads)^2^. Connectivity can be characterised in structural and functional terms: structural connectivity measures habitat contiguity and is dependent on the landscape structure independently of any attributes of organisms moving through the landscape space; functional connectivity explicitly measures habitat contiguity with reference to the attribute of a particular organism (or group of organisms) moving through the landscape space^3^. Structural connectivity is typically markedly easier to measure, and has been the focus of the vast majority of attempts to characterise the connectivity of natural/semi-natural habitat in cities and towns^2^,^4^,^5^,^6^,^7^. How well this reflects connectivity in urban systems from an organismal perspective or with regard to ecosystem function remains largely unknown, although it is plain that structural connectivity can both under- and over-estimate levels of functional connectivity. Indeed, the widespread impression that urbanisation leads to low connectivity is doubtless a gross simplification.

Studies of connectivity of natural and semi-natural habitat in cities and towns have also almost exclusively focused on measuring this from a two-dimensional (2D) perspective, for example, using optical remote sensing data to map the distribution of plants in the landscape. Whilst not unusual in other kinds of landscapes, this may be particularly problematic in urban systems because of the heterogeneity in vegetation canopy complexity, both spatially and volumetrically. Even small areas of greenspace (e.g. individual public parks, domestic gardens) may comprise a wide diversity of vegetation types in close proximity to each other^8^,^9^,^10^, and the vertical distribution of that greenspace is important in governing the connectivity between patches. Many organisms do not move in 2D space but utilise one or more strata of the vegetation, therefore understanding this variation will be critical to quantifying connectivity at a species or ecosystem service provision level.

Recent studies^11^,^12^ have started to characterize and describe the three-dimensional (3D) spatial structure and arrangement of vegetation in cities using light detection and ranging technology (lidar) but have not considered 3D connectivity. The paucity of studies of the 3D structure and connectivity of green habitat in cities and towns^13^ is understandable given the fine-grained structure of this habitat, the spatial heterogeneity, the technical challenges of disentangling the 3D structure of vegetation from that of built infrastructure, and the scarcity of data and methods for describing the 3D organisation of greenspace components at a sufficient extent and fine grain size that allows the spatial heterogeneity to be captured^14^,^15^,^16^. Here we employ state-of-the-art waveform laser scanning technology to measure the horizontal and vertical distribution of greenspace elements across three UK towns, so as to test how a 3D greenspace perspective compares with 2D measures of connectivity. The study represents a snapshot in time, but the three towns exhibit variable urban forms, structures and greenspace patterns, while collectively displaying a broader range of conditions than could be found by studying one site in isolation. We tested the hypothesis that spatial metrics describing structural and functional connectivity will be higher (i.e. indicating a more connected landscape) when using 2D greenspace data as compared to those obtained when grass, shrub and tree layers are disaggregated using 3D waveform lidar data.

## Methodology

### Study area

This study was conducted in the ‘Cranfield triangle’, a region in southern England, UK (52°07′N, 0°61′W), comprising the three adjacent towns of Milton Keynes, Luton, and Bedford (Supplementary Figure S1). These each have similar topographical, climatic and human population sizes (respectively, c. 230,00, c. 240,000, and c. 160,000; 2011 Census, UK), but different historical backgrounds and thus, they exhibit variability in patterns of architectural and green features^17^. Milton Keynes was planned as new town in the 1960s and designed with green connectivity in mind^17^. Bedford is a smaller county town and includes typical patterning of clustered housing and greenspace arising from a medieval layout^17^. Luton is representative of Victorian terraced urbanism and includes large industrial areas^17^.

### Input data and processing 2D and 3D green surface maps

Waveform lidar and hyperspectral data were collected during four overflights by the Natural Environment Research Council Airborne Research and Survey Facility (NERC ARSF) between June and September 2012. The aircraft was carrying a lidar sensor (Leica ALS50-II) able to capture full-waveform data^18^,^19^ and an “Eagle” spectrometer covering the electromagnetic spectrum from 407 nm to 1007.10 nm in 253 separate wavebands and at a 2 m grid resolution (with the exception of a small area of Luton where the hyperspectral Eagle data were captured at 4 m spatial resolution due to flying height restrictions near a major commercial airport).

A 2D spatial model describing the distribution of green surfaces was derived from the Eagle spectrometer, yielding a 2D map layer showing where vegetation was present. Vegetated areas were differentiated from those with no vegetation using the Normalized Difference Vegetation Index (NDVI)^20^ based on reflectance in the visible range (mean reflectance values from 500.84 nm–679.70 nm) and near infra-red (mean reflectance of 761.21 nm–960.78 nm). In the final model a threshold of NDVI ≥ 0.2 was selected from histograms^21^ and was used to define the presence of vegetation.

Waveform lidar data require pre-processing to correct for a variety of effects accurately to estimate vegetation cover. A comprehensive approach to handle these followed the methods outlined in Hancock *et al*.^11^,^18^. To evaluate the impact of vertical stratification of greenspace on connectivity measures, we generated three separate vertical strata layers from the waveform lidar data. First, the waveform lidar data were converted into a voxel (volumetric pixel) map of fractional cover at 1.5 m by 1.5 m horizontal and 50 cm vertical resolution, following the method presented in Hancock *et al*.^11^. This was combined with the NDVI derived vegetation map to exclude non-vegetated areas. The presence/absence of vegetation in voxels was determined by setting a 1% cover threshold; voxels containing 1% or more of vegetation were marked as vegetated and those with less were marked as empty. From these 3D voxel results we distinguished three key vegetation strata (and by virtue of the capabilities of waveform lidar were also able to quantify understorey coverage): (i) Grass, NDVI ≥ 0.2 and height < 0.5 m; (ii) Shrubs, NDVI ≥ 0.2 and height between 0.5 and 4 m; and (iii) Trees, NDVI ≥ 0.2 and height > 4 m; and also (iv) Not green, NDVI < 0.2. Finally we projected the 3D voxel presence of each stratum into its corresponding 2D surface projection on the ground and quantified the surface cover for each stratum individually.

**Figure 1:**
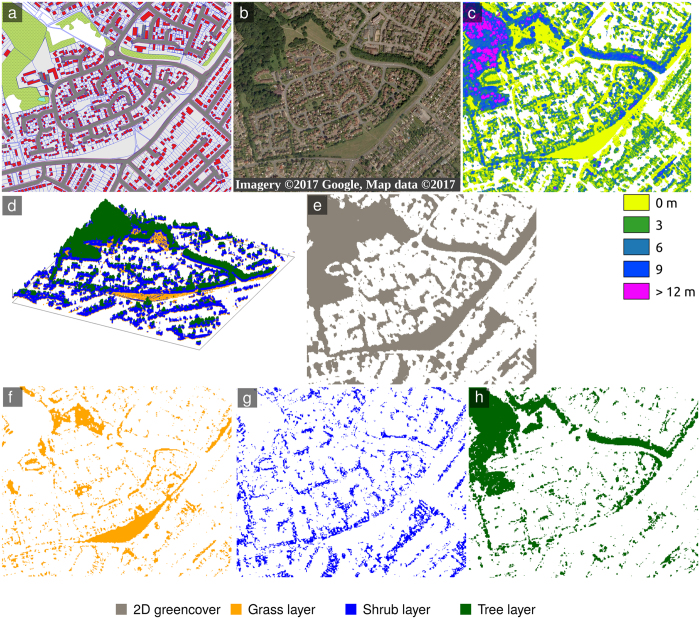
A detailed view of an area of the study in Luton showing the different data sources: (**a**) distribution of buildings (red) and roads (grey). Map (**a**) ‘Contains Ordnance Survey data © Crown copyright and database right 2013’; (**b**) Aerial image from Google Earth ‘Imagery ©2017 Google, Map data ©2017’; (**c**) Waveform lidar data visualization on a 2D plane from the top canopy showing major classes of varying vegetation height; (**d**) Waveform lidar data visualization as point clouds on a 3D plane classified according to the legend shown at the base of the figure; (**e**) 2D greenspace map derived from NDVI showing the location of ‘green’ (grey pixels) and non-green (white pixels) areas; and (**f**), (**g**) and (**h**) show the distribution of the vegetation layers derived from the 3D waveform lidar data: (**f**) grass, (**g**) shrubs and (**h**) trees. In this study we compared connectivity from maps (**e**), (**f**), (**g**) and (**h**). Maps generated using QGIS software version 2.14^25^,^26^.

These steps resulted in an overall 2D vegetation map derived from the NDVI, showing the location of urban greenspaces, and a series of layers derived from the 3D voxel analysis, showing single vegetation maps per stratum: one 2D map showed the location of grass areas, another shrubs and a third trees (Fig. 1).

### Structural connectivity metrics

There are a vast number of software-driven approaches to calculating structural connectivity^22^. Our aim was not to compare these different approaches, but rather to use appropriate metrics to quantify the differences between the 2D-derived and 3D-derived connectivity measures within three urban forms. We selected the following metrics which allow direct comparison between areas with varying size and form: (i) Landscape proportion - the proportion of landscape covered by vegetation; (ii) Small patch density - the number of disjunct patches smaller than 30 m^2^ per total landscape area. Using this grain size, it was possible to capture detail of private gardens and small green features that are often overlooked in other greenspace studies reliant on satellite data with coarser spatial resolution (e.g. Landsat data have 30 m spatial resolution); (iii) Largest patch index - the percentage of the landscape comprised of the largest greenspace patch; and (iv) Connectivity Index (CI) or landscape division index^23^, 
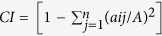
, where *aij* = area (m^2^) of patch *ij*; *A* = total landscape area (m^2^) - the probability that two randomly chosen pixels in the landscape are not situated in the same patch of the corresponding patch type. The higher the index, the lower connectivity within a surface landscape.

### Functional Connectivity

We computed the CI for every 1 m buffer increment of the original vegetated surfaces (for layers shown in Fig. 1e–h) until the buffer size was large enough to compose a completely connected surface (i.e. a fully vegetated map). In the resulting models, buffer increments represent the dispersal capacity or mobility of organisms between green patches – this is therefore not structural connectivity but a functional indicator related to organismal mobility characteristics. We plotted the buffer increment size (*x*) against CI (*y*) to model the functional connectivity in different urban forms. We then computed the difference between 2D CI (calculated from Fig. 1c) and the corresponding CI in 3D derived strata (Fig. 1d–f) within grass, shrubs and tree layers, at varying distances.

### Computational implementation

Voxel data were generated using voxelate.c program available at (https://bitbucket.org/StevenHancock/voxelate). NDVI and vegetation stratification maps where generated using GRASS GIS software version 6.4^24^. Structural connectivity metrics where computed using LecoS – Landscape Ecology Statistics plugin version 2.6 under QGIS software version 2.14^25^. Functional connectivity buffers were generated in GRASS GIS software version 6.4^24^ and CI was computed as for structural connectivity but using a Python version 2.7.12 scripting routines. Graphics were generated using R software version 3.2.3^26^. All code for data processing are available at the web repository: https://github.com/stefano-cornwall/urbanConnect.

## Results

### Structural connectivity

**Figure 2:**
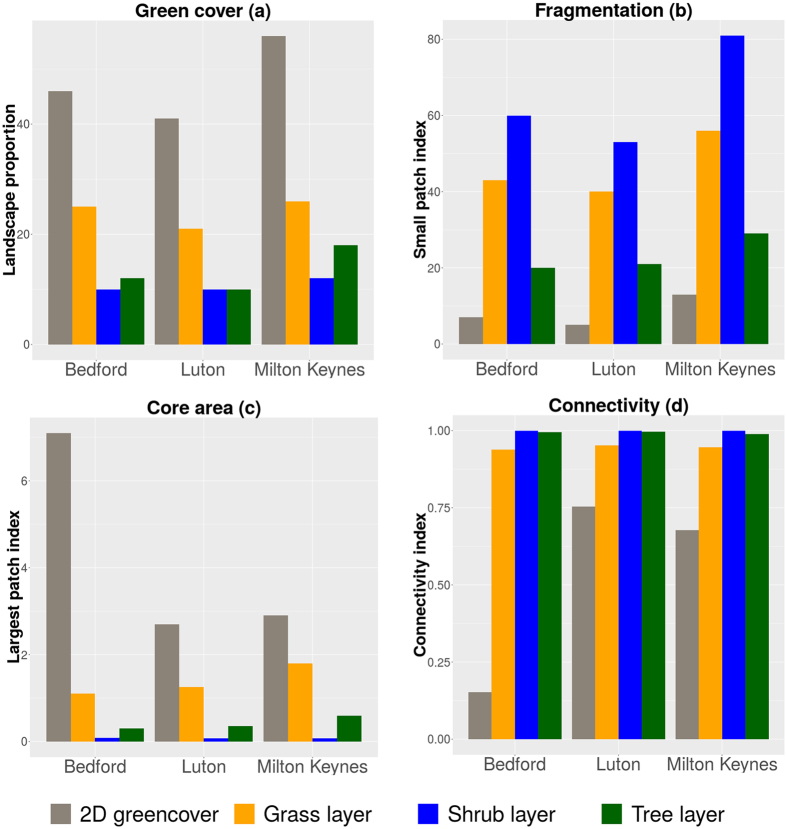
Structural connectivity parameters for different connectivity metrics for the three towns derived from analysis of different 3D structural layers (coloured bars) and 2D green cover (grey). (**a**) Proportion of green surface cover - where higher values of the landscape proportion parameter represent greater overall amounts of green cover; (**b**) fragmentation of green patches <30 m^2^ – where higher values of the small patch index parameter correspond to a more fragmented landscape; (**c**) core area cover – where higher values of the largest patch index parameter correspond to a higher percentage of the landscape comprised of the largest greenspace patch; (**d**) CI or the probability that two random chosen locations in a green surface are not connected – where the higher CI parameter corresponds to a lower overall spatial connectivity.

When comparing the 3D derived strata and 2D analysis, the structural connectivity measures varied as shown in Fig. 2. Key patterns and distinctions as found for all three towns were: (i) 2D metrics indicated a greener landscape than 3D derived strata (Fig. 2a shows a higher percentage of green landscape in 2D than in any of the 3D derived strata); (ii) 2D metrics indicated a less fragmented landscape than 3D derived strata (Fig. 2b shows a higher density of patches smaller than 30 m^2^); (iii) A wider core connected zone was indicated from 2D data as compared to 3D derived strata, as a proportion of the overall area (Fig. 2c shows the largest patch index in the 2D analysis); (iv) A more connected 2D surface as compared to the 3D derived strata (Fig. 2d shows lower CI values from 2D analysis implying higher structural connectivity) - according to 2D metrics, Bedford had the highest structural connectivity (Fig. 2d).

Analysing the differences in the individual structural connectivity data for the 3D derived strata (Fig. 2), in all three towns grass occupied the highest proportion of green space, followed by trees and then shrubs in Bedford and Milton Keynes; in Luton trees and shrubs occurred in equal proportions (Fig. 2a). At the stratum level, we also found the shrub stratum to be more fragmented than trees or grass (Fig. 2b) and with a smaller patch index (Fig. 2c). This means that shrub-covered areas in the towns were more sparsely arranged in space than grass or trees, which evidently formed areas of more continuous cover.

Trees showed the opposite behaviour to shrubs, being less fragmented than grass or shrubs (Fig. 2b) and with a larger patch index than shrubs but not grass (Fig. 2c). Finally, the grass stratum had an intermediate level of fragmentation between trees and shrubs (Fig. 2b) but the highest patch index of the 3D derived strata (Fig. 2c). Looking at the CI data (Fig. 2d) there were consistent connectivity patterns in the 3D derived strata – i.e. that grass was always better connected than trees, which were better connected than shrubs (lower CI means higher structural connectivity in Fig. 2d).

Looking at all layers, Milton Keynes had a more complex structure of urban vegetation than Luton and Bedford (i.e. higher fragmentation in Fig. 2b).

### Functional connectivity

**Figure 3:**
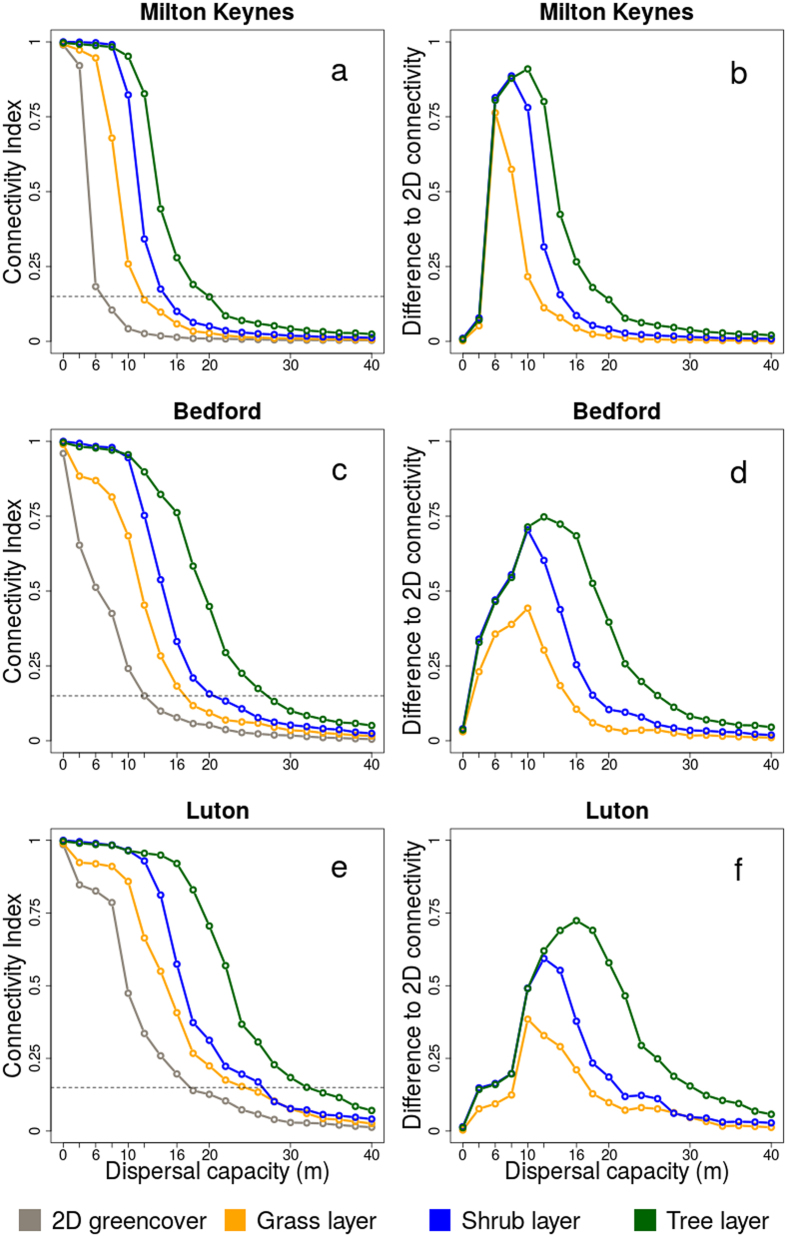
Models of functional connectivity in vegetation given different dispersal capacities or mobilities derived from 3D structural layers (coloured lines) and 2D green cover (grey) within three towns in the UK are shown in panels (**a**), (**c**), and (**e**). The difference in functional connectivity from the 2D perspective is shown for the same three towns in panels (**b**) (**d**) and (**f**) for the grass (yellow), shrub (blue) and tree (green) layers, derived from the 3D waveform lidar data. 3D analyses include vertical movements within individual vegetation layers while the 2D vegetation perspective merges all layers and is unable to differentiate structure and vertical movements within strata. The dotted lines highlight thresholds of CI < 0.15 denoting high levels of connectivity.

Figure 3 shows two plots for each town. On the left hand side is a straightforward plotting of functional CI against dispersal capacity, whilst on the right hand side is plotted the difference between the 3D derived strata functional connectivity and the corresponding 2D measure at varying dispersal capacities. These results reveal increasing functional connectivity from trees to shrubs, shrubs to grass and between grass and the 2D perspective (Fig. 3) for all three study areas. In all three towns, all layers become completely functionally connected (with CI approaching zero) at a dispersal distance of 40 m (note: Milton Keynes have higher functional connectivity, CI approaches zero at a lower dispersal distance of ~30 m).

The analysis of the 3D derived strata (Fig. 3) showed that grass functional connectivity occurred at different distances in the three towns. Using a threshold of CI < 0.15, representing high levels of connectivity (i.e. probability > 85% for two random patches to be connected), this distance was 12 m in Milton Keynes, 18 m in Bedford and 26 m in Luton. Using the same threshold, functional connectivity for shrubs was achieved at 20 m in Milton Keynes, 22 m in Bedford and 28 m in Luton. Tree canopy functional connectivity occurred at 20 m in Milton Keynes, 28 m in Bedford and 32 m in Luton. Within the 2D analysis, the same measure of functional connectivity was much greater at shorter distances (8 m for Milton Keynes, 12 m for Bedford and 18 m for Luton). Overall, high levels of functional connectivity occurred at increased distances from the 2D greencover to the grass, shrubs and tree layers respectively and Milton Keynes had higher levels of connectivity compared to Luton and Bedford.

We found different functional CI patterns according to the 2D and 3D derived vegetation layer considered: discrepancies (Fig. 3) were found to be higher at low dispersal capacity, and absolute discrepancies were higher in trees than shrubs and grass. We found all of these patterns to be repeated at all three study sites (Fig. 3). The biggest differences between 2D functional connectivity and connectivity within each of the 3D derived strata were found to occur at distance ranges of between 6 m (Fig. 3b shows this to be the distance of the peak of difference in the grass layer data for Milton Keynes) and 16 m (Fig. 3f shows the peak of difference in the tree layer for Luton) dispersal capacity. For example, the 2D perspective in Milton Keynes showed highly connected surfaces at 6 m (CI = 0.183) as compared to individual strata having low connectivity at the same distance (CI grass = 0.946; CI shrubs = 0.997; CI trees = 0.988). Similar discrepancies between 2D CI measures and 3D derived strata CI measures were evident in all three towns. The distance at which the ‘difference’ plots peaked (Fig. 3b,d,f) showed a regular pattern with respect to grass, trees and shrubs but also evidenced the scale-dependent and site-dependent differences between 2D and 3D derived CI.

## Discussion

Urban greenspace is heterogenous and structured. It exhibits both 2D and 3D spatial complexity. Despite this, due to the inherent difficulties of calculating 3D complexity, many research projects have focused on analysing the connectivity of urban greenspace using basic 2D greenspace estimates mostly determined from standard optical and infra-red remote sensing observations of photosynthetic indicators, particularly NDVI^27^,^28^,^29^,^30^,^31^. Such measures neglect to capture the vertical organisation of the green material in the urban volume. In using these 2D spatially distributed estimates of urban greenness, one is making the assumption that the greenspace is uniformly distributed in the volume – an assumption we have shown (Fig. 1), unsurprisingly, to be flawed. NDVI allows mapping presence/absence of vegetation but conveys nothing about the vertical distribution, which is important in urban areas where one or more layers may overlap. In using waveform lidar – a technique that allows the green volume to be calculated within individual, vertical vegetation layers, we have been able to reveal for the first time the impact of this 2D greenspace bias on connectivity estimates. Our models of functional connectivity are spatial models that could be applied to different organisms knowing their dispersal/movement range capacity and the vegetation strata they use to move.

Specifically, this work has shown that relying on optical 2D greenspace data (NDVI) results in a positively biased estimate of greenspace connectivity estimates and that this is true irrespective of urban form, from compact urban spaces (i.e. Bedford) to areas with designed greenspace (i.e. Milton Keynes). This is particularly critical to consider when vertical strata form barriers in space, exerting a disproportionate effect on organismal movements or ecosystem service provision (for example, lines of trees that filter air pollution and provide important barriers for noise reduction)^32^ or roads that hinder movement of animals through urban spaces^33^,^34^. Further supporting this is our demonstration of the discrepancies in connectivity patterns between 2D and 3D derived strata layers at short distances (Fig. 3) of 6 m, 12 m and 16 m respectively for Milton Keynes, Bedford and Luton. While the 2D perspective shows connected surfaces at these short distances, the 3D derived perspective does not.

Urban ecosystems have numerous spatial barriers to ecological movement, and these barriers clearly increase when considering the vertical distribution of greenspace in a 3D landscape. For example, we show that organisms that rely solely on trees for movement will have reduced connectivity relative to those that rely solely on grass. NDVI fails to capture this variation, while a 3D derived analysis, as we have demonstrated with waveform lidar can deliver such understanding successfully. Further, the 3D landscape also determines permeability of urban structures such as buildings and walls, which at the lower strata will halt connectivity for many organisms, but may facilitate it for others depending on the strata the organism relies on for movement.

Of course the level of bias between 2D- and 3D-derived measures of connectivity will vary with the ecological niche of the organism in question. Organisms such as grey squirrels (*Sciurus carolinensis*) that are common in urban areas in the UK move through all three strata considered here (Fig. 1e: 2D perspective from NDVI), and may be less restricted in their connectivity than those such as European hedgehogs (*Erinaceus europaeus;* occasional visitors to urban gardens) that are confined to specific levels (Fig. 1f–h. derived from 3D perspective). Here we model the 3D spatial distribution of grass, shrubs and trees, however we do not consider the relative importance of the different strata in facilitating connectivity. For example, large trees are keystone features in modified landscapes for facilitating connectivity for a wide range of organisms and so may be disproportionately more important for connectivity for many organisms^35^,^36^. Future research needs to move forward to determine the relative contribution of each stratum and their combined influence on connectivity of individual organisms. There is also significant seasonal variation in connectivity in the urban landscape for some organisms^30^, because phenology varies across the strata connectivity is likely to show greater temporal variation in 3D over 2D greenspace.

This study represents the first in a new generation of high resolution, data-driven spatial techniques that model the 3D landscape. In summary, we found that when 3D stratification was omitted it resulted in an overestimation of connectivity (Figs 2d and 3b,d,f), and that landscapes with more complex 3D vegetation structure (fragmentation in Fig. 2b) have greater functional connectivity (Fig. 3a,c). Unsurprisingly, Milton Keynes, a ‘green town’ designed with connectivity in mind, was found to have the best functional connectivity (Fig. 3). We conclude on the importance of considering the vertical stratification of the vegetation in urban systems to understand patterns of landscape connectivity which are strategic for low mobility organisms and for the provision of urban ecosystem services.

## Additional Information

**How to cite this article:** Casalegno, S. *et al*. Ecological connectivity in the three-dimensional urban green volume using waveform airborne lidar. *Sci. Rep.* **7**, 45571; doi: 10.1038/srep45571 (2017).

**Publisher's note:** Springer Nature remains neutral with regard to jurisdictional claims in published maps and institutional affiliations.

## Supplementary Material

Supplementary Information
