# Supplementary material for: Ecological connectivity in the three-dimensional urban green volume using waveform airborne lidar
Source: Sci Rep. 2017 Apr 6;7:45571. doi: 10.1038/srep45571 (PMC5382541; doi:10.1038/srep45571)
Supplement: Supplementary Information [file srep45571-s1.pdf]

Supplementary Figure S1

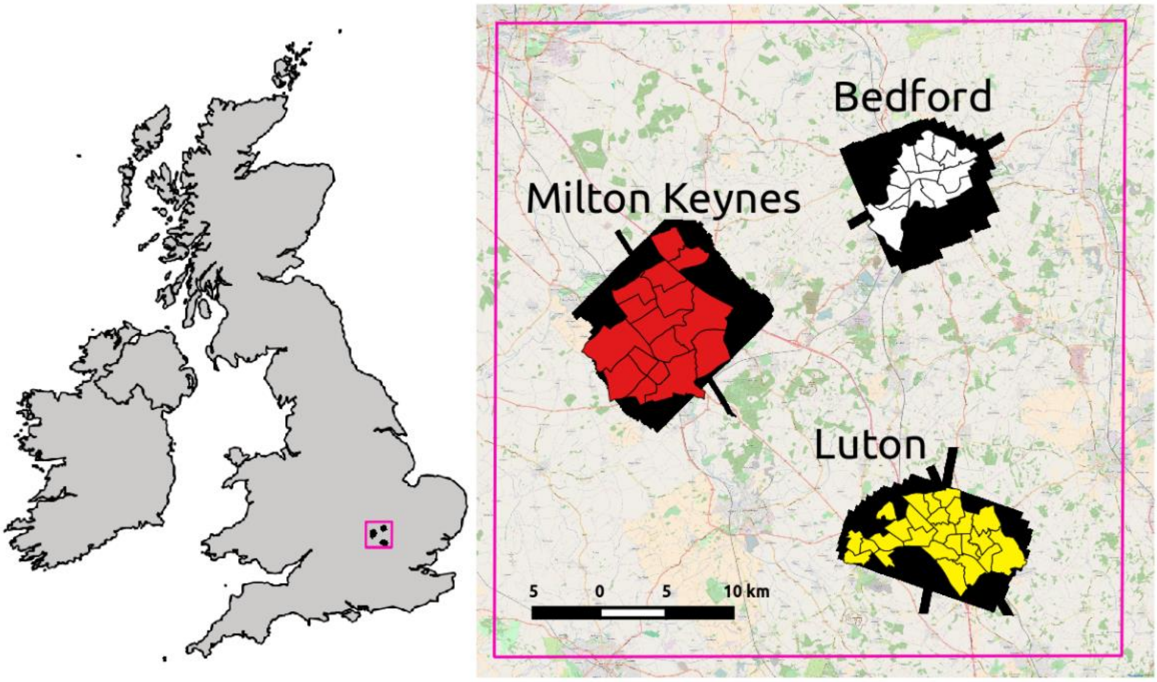

Location of the overall study area within the United Kingdom (left) and details of the three towns considered in this study (right). The black zones in the right panel define the flight missions on which the waveform lidar data and NDVI data were collected; the areas coloured in white (Bedford), red (Milton Keynes) and yellow (Luton) highlight the town administrative boundaries. Maps generated using Qgis software [QGIS Development Team. QGIS Geographic Information System. Software version 2.14. Open Source Geospatial Foundation Project. http://www.qgis.org/ (2016)].
